# '*Candidatus* Megaira' are diverse symbionts of algae and ciliates with the potential for defensive symbiosis

**DOI:** 10.1099/mgen.0.000950

**Published:** 2023-03-10

**Authors:** Helen Rebecca Davison, Gregory D. D. Hurst, Stefanos Siozios

**Affiliations:** ^1^​ Institute of Infection, Veterinary and Ecological Sciences, University of Liverpool, Crown Street, Liverpool L69 7ZB, UK

**Keywords:** symbiosis, bacteria, *Rickettsiales*, algae, ciliates, microeukaryotes

## Abstract

Symbiotic microbes from the genus *'Candidatus* Megaira' (* Rickettsiales *) are known to be common associates of algae and ciliates. However, genomic resources for these bacteria are scarce, limiting our understanding of their diversity and biology. We therefore utilize Sequence Read Archive and metagenomic assemblies to explore the diversity of this genus. We successfully extract four draft '*Ca*. Megaira' genomes including one complete scaffold for a '*Ca*. Megaira*'* and identify an additional 14 draft genomes from uncategorized environmental metagenome-assembled genomes. We use this information to resolve the phylogeny for the hyper-diverse '*Ca*. Megaira', with hosts broadly spanning ciliates, and micro- and macro-algae, and find that the current single genus designation '*Ca*. Megaira' significantly underestimates their diversity. We also evaluate the metabolic potential and diversity of *''Ca*. Megaira' from this new genomic data and find no clear evidence of nutritional symbiosis. In contrast, we hypothesize a potential for defensive symbiosis in *'Ca*. Megaira*'*. Intriguingly, one symbiont genome revealed a proliferation of ORFs with ankyrin, tetratricopeptide and leucine-rich repeats such as those observed in the genus *Wolbachia* where they are considered important for host–symbiont protein–protein interactions. Onward research should investigate the phenotypic interactions between *'Ca*. Megaira*'* and their various potential hosts, including the economically important *Nemacystus decipiens*, and target acquisition of genomic information to reflect the diversity of this massively variable group.

## Data Summary

Genomes assembled in this project have been deposited in bioproject PRJNA867165. Supplementary tables and figures have been submitted to the Microbiology Society Figshare under manuscript number MGEN-D-22–00320. Supplementary data for Supplementary interactive version of Figure 7, Figs S1–S3 and Tables S1–S10 (available in the online version of this article) are also available in this Figshare collection: https://doi.org/10.6084/m9.figshare.c.6213559.v2 [1].

**Impact Statement**

Bacteria that live inside larger organisms commonly form symbiotic relationships that impact the host's biology in fundamental ways, such as improving defences against natural enemies or altering host reproduction. Certain groups such as ciliates and algae are known to host symbiotic bacteria commonly, but our knowledge of their symbiont's evolution and function is limited. One such bacteria is *'Candidatus* Megaira*'*, a *Rickettsiales* that was first identified in ciliates, then later in algae. To improve the available data for this common but understudied group, we searched the genomes of potential hosts on online databases for *Rickettsiales* and assembled their genomes. We found four *'Ca*. Megaira*'* in this way and then used these to find a further 14 genomes in environmental metagenomic data. Overall, we increased the number of known *'Ca*. Megaira*'* draft genomes from two to 20. These new genomes show us that *'Ca*. Megaira*'* is far more diverse than previously thought and that it is potentially involved in defensive symbioses. In addition, one genome shows striking resemblance to a well-characterized symbiont, *Wolbachia *, in encoding many proteins predicted to interact directly with host proteins. The genomes we have identified and examined here provide baseline resources for future work investigating the real-world interactions between the hyper-diverse *'Ca*. Megaira*'* and its various potential hosts, such as the economically important *Nemacystus decipiens*.

## Introduction

A wide range of bacterial species reside as endosymbionts in both microeukaryotes and algae [2–9]. Symbiont presence can affect the biology of their host in significant ways, from reproductive manipulation [10, 11] to stress tolerance [12], nutrient production [13, 14] and methanogenesis [15]. Symbionts in microeukaryotes were recognized as early as 1902 in the amoeba *Pelomyxa* [16]. Whilst some systems are relatively well understood, such as *Caedibacter* and *Paracaedibacter* in *Paramecium* [11], our knowledge of symbiont evolution and function in microeukaryotes is fragmented in comparison to symbioses in animals and terrestrial plants. For instance, the effects of endosymbiotic bacteria in algae are currently unknown, with studies rarely extending beyond the presence of the symbioses and the phylogenetic affiliation of the symbiont [8, 9, 17].

In the last decade, *Rickettsiales* have been identified as a group that commonly form symbioses with microeukaryotes as well as invertebrates and algae [5, 18–24]. The origins of some families within the *Rickettsiales *, such as the *Rickettsiaceae *, may derive from symbioses with microeukaryotes [21]. *'Candidatus* Megaira*'* is a member of *Rickettsiales* and a relative of '*Ca*. Tisiphia', *Rickettsia* and *Wolbachia,* which are prolific endosymbionts with wide-ranging effects on their hosts [25–30]. As such, *'Ca*. Megaira*'* has the potential to impact its hosts in many ways. In the few functional studies that have been completed, the presence of*'Ca*. Megaira*'* was shown to improve growth in some *Paramecium* [20, 31]. However, in contrast to *Rickettsia* and *'Ca*. Tisiphia', there is currently very limited genomic data for *'Ca*. Megaira*'*, with a single closed and a single draft genome, both from algae [32].

The increasing power and reliability of bioinformatic tools now enable us to extract high-quality microbial symbiont genomes from the Sequence Read Archive (SRA) deposits [32, 33]. We can search for symbiotic bacteria in hosts without *a priori* hypotheses to establish novel symbiotic interactions with target microbes, and then assemble draft genome sequences for the symbionts. Declining costs have driven a surge in sequencing non-model taxa such as microeukaryotes and environmental DNA, providing ample data from which to extract symbiont genomic data. For taxa such as *'Ca*. Megaira*'* where there is little genomic information available, these data then provide us with the opportunity to explore their evolution and diversity in more detail and generate hypotheses as to the function of the symbioses found.

In this study, we search for and extract potential *'Ca*. Megaira*'* symbionts in GenBank SRA data for ciliates and all current classifications of micro- and macro-algae. In addition, we identify *'Ca*. Megaira*'* genomes amongst publicly available metagenome-assembled genomes (MAGs) in GenBank. These data collectively expand the known whole genomes of *'Ca*. Megaira*'* from two to 20 and enable phylogenomic and metabolic analyses.

## Methods

### Collection of external genomes for metagenomics and phylogenomics

Illumina SRA data for all ciliates and current classifications of Algae as of 5 May 2021 were downloaded from NCBI to screen for symbiont genomes. These were*: Bacillariophyceae, Charophyceae, Chlorarachniophytes, Chlorophyceae, Chlorophyta, Chrysophyceae, Cillophora, Cryptophyceae, Dictyochophyceae, Dinophyceae, Euglenophyceae, Eustigmatophyceae, Haptophyta, Mesostigmatophyceae, Phaeophyceae, Rhodophyta, Synurophyceae, Ulvophyceae* and *Ulvophyceae*. Libraries were excluded if they were: extremely shallow sequencing efforts below 500 Mb, macronucleus-only sequencing, mutant resequencing, listed as antibiotic treated or ddRAD sequence. In total 1113 of 3445 algae and 464 of 547 ciliate libraries were identified for onward analysis.

### Metagenomic identification, assembly of genomes and phylogenomic analysis

SRA deposits were screened for the presence of *Rickettsiales* using Phyloflash [34]. *Rickettsiales *-positive libraries were taken forward for metagenomic assembly and binning to extract full genome sequences as described in Davison *et al*. [32]. Briefly, metagenomic assembly, binning and quality check were performed with Megahit, Metabat2 and CheckM [32, 35–38]. Samples that contained >50 % complete symbiont genomes with <5 % contamination were taken forward for further examination and manual refinement. GTDBtk [39] was used for taxonomic classification of each extracted genome and to identify their nearest relatives. Genome bins identified as *Rickettsiales* were named as follows: first three letters of their closest relative + first letter of host genus + first four letters of host species + bin number. For example, a *'Ca*. Megaira*'* from a *Mesostigma viride* SRA in bin 4 would be labelled MegMviri4.

*Nemacystus decipiens* (bioproject PRJDB7493) had multiple SRA libraries from the same biosample which we co-assembled with Megahit. Then, each library was individually mapped back to the assembly with bowtie2 [40] and symbiont bins were identified with Metabat2. Nine of the libraries were mate-pair reads with insert sizes ranges from 2 to 13 kb and were used to scaffold the draft assembly and close the genome using the BESST algorithm [41].

Additional putative *'Ca*. Megaira*'* genomes were identified on GenBank as follows. We performed blastp searches of core '*Ca*. Megaira' proteins from our new draft genomes to identify homologues in the non-redundant protein sequence database using default settings [42]. Amongst the top hits were protein sequences from 14 existing but unclassified environmental MAGs. These MAGs were retrieved and their affiliation to '*Ca*. Megaira' was confirmed using the GTDBtk database [42].

In order to anchor our genomes against previous knowledge of *'Ca*. Megaira*'* diversity, 16S rRNA gene sequences were assembled for *'Ca*. Megaira*'* symbionts where possible. Although, due to the limitations of metagenomic binning and assembly, 16S rRNA retrieval was not possible for several environmental metagenomes. MegHsini1 is a partial genome and two 16S rRNA sequences can be extracted with Anvi'o 7 [43]. The most complete of these was used for 16S rRNA sequence placement. The least complete one seems to be related to *Deineraceae* and was deemed a probable contaminant. Additional sequences can be found in Table S7.

The draft genome data were used to enable a phylogenomic approach to *'Ca*. Megaira*'* diversity alongside existing known *'Ca*. Megaira' genomes (Tables S1 and Fig. S2). Orthologous genes across the 20 *'Ca*. Megaira*'* genomes were identified using Anvi'o 7 [43] for the purpose of extracting the core gene clusters (50 gene clusters). Average nucleotide identity (ANI) was calculated through pyANI within Anvi'o 7 (Table S4). Average amino-acid identity (AAI) was calculated pairwise for each genome pair through the AAI-Matrix calculator from the enveomics toolbox (Table S3) [44]. Synteny between JAFLDA01 and MegNEIS296 was established with PROmer in the MUMmer3 package with default settings [45]. Maximum-likelihood trees were produced with IQ-Tree and automatic best model selection using ModelFinder [46, 47] with 1000 replicates of UltraFast Boostrap [48] and the SH-like Approximate Likelihood Ratio Test [49]. Models selected for each tree were as follows: *'Ca*. Megaira*'* core amino acids=LG+F+I+G4, and *'Ca*. Megaira*'* 16S rRNA=GTR+F+R3. Bayesian phylogenetic inference was performed in Phylobayes-mpi [50] and the CAT-GTR model. Two independent chains were run in parallel for at least 40 000 cycles each until convergence was observed (maxdiff <0.1).

### Examining metabolic potential, annotation and identifying NRPS systems

High-quality genomes from the above were defined as >90 % complete and contamination <10 %. This process defined two existing *'Ca*. Megaira*'* genomes (MegCarteria and MegNEIS296), three novel genomes derived from the SRA (MegSroe9, MegMviri4 and MegNdeciBESST), and five novel genomes derived from MAGs (JAFLDA01, VGEX01, JAJTEJ01, NVVL01 and JAFLCZ01) as high quality, and these were analysed alongside a *'Ca*. Tisiphia' genome and *Orientia *. Metabolic potential was predicted based on KEGG annotations by Anvi'o 7 [43, 51]. Heatmaps of pathway completeness were sorted by phylogeny and plotted in Python with Seaborn [52, 53]. An upsetplot of shared gene clusters between genomes was constructed with ComplexUpset [54] in R 4.1.0 [55].

AntiSMASH [56] was then used on the eight high-quality genomes to predict secondary metabolites such as those produced by the non-ribosomal peptide synthetase (NRPS) systems. These have been identified previously in the existing *'Ca*. Megaira*'* genome, MegNEIS296 (ASM2041082v1). Clinker was used to visualize the similarity between the resulting systems found [57]. Further annotations were made with InterProScan 5 [58] using Pfam, TIGRFAM, PANTHER and GOterms.

## Results

### Assembly of genomes

After metagenomic binning, four SRA deposits were identified as harbouring *'Ca*. Megaira*'* and taken forward for further analysis. All but one genome is >90 % complete according to checkM results (Table 1). MegHsini1 is derived from a single cell genomics approach and was just 62.84 % complete and thus not included in onward metabolic analyses, but core gene clusters and marker genes were retained for phylogenetic placement. No *Rickettsiales* other than *'Ca*. Megaira*'* were recovered. The *'Ca*. Megaira*'* from *Nemacystus decipiens* (PRJDB7493) was the only genome that could be assembled into one scaffold, albeit not closed, using the available mate-pair data. This genome, named here as MegNdeciBESST, has a total size of about 1.3 Mb and contains 20 gaps, ranging from 346 to 5679 bp. Fourteen additional environmental MAGs, previously characterized as unclassified *Rickettsiales *, were identified in GenBank. These environmental MAGs are of similar quality as the MAGs constructed from SRA databases here (Table 1).

**Table 1.** Genome statistics and sources; in-depth metadata including SRA sample accessions can be found in Table S1

Name	Bacteria accession no.	Clade	Source accession	Host type	Source	CheckM completion score	CheckM contamination score	Genome size	No. of contigs	GC content (%)	Completion status
**Genomes assembled in this study**											
MegNdeciBESST	SAMN30190846	n/a	PRJDB7493	Algae	*Nemacystus decipiens*	96.21	0.71	1273930	23	31.75	Single scaffold
MegMviri4	SAMN30190847	*'Candidatus* Megaira', Clade A	PRJNA517804	Algae	*Mesostigma viride*	96.21	3.32	1410865	28	33.66	Contigs
MegSroe9	SAMN30190848	*'Candidatus* Megaira', Clade A	PRJNA507905	Ciliate	*Stentor roeselii* strain: QDSR01	95.50	1.94	1258451	82	33.65	Contigs
MegHsini1	SAMN30190849	n/a	PRJNA546036	Ciliate	*Hartmannula sinica*	62.84	0.95	702013	183	28.35	Contigs
**Existing unclassified MAGs**											
RFMR01	GCA_009927585.1	*'Candidatus* Megaira', Clade A	PRJNA495371	Unknown	Freshwater	86.63	3.12	1145548	209	33.63	Contigs
RGPV01	GCA_010026065.1	*'Candidatus* Megaira', Clade A	PRJNA495371	Unknown	Freshwater	62.76	14.26	2044025	736	33.78	Contigs
RGWT01	GCA_010029695.1	'Candidatus Megaira', Clade A	PRJNA495371	Unknown	Freshwater	54.07	4.55	1011306	546	34.82	Contigs
JAFLCZ01	GCA_017302665.1	'*Candidatus* Megaira', Clade A	PRJNA704939	Unknown	Activated sludge	98.58	7.11	1646433	29	33	Contigs
JAGOTB01	GCA_018062005.1	'Candidatus Megaira', Clade A	PRJNA524094	Unknown	Wastewater	76.13	6.79	1346348	220	33.5	Contigs
JAGWWU01	GCA_018970295.1	'*Candidatus* Megaira', Clade A	PRJNA675967	Unknown	Mine drainage	87.68	4.74	1251243	73	33.5	Contigs
JAJTEJ01	GCA_021300375.1	'*Candidatus* Megaira', Clade A	PRJNA464361	Unknown	Lake water	95.34	2.84	1300143	76	33.5	Contigs
VGEX01	GCA_016869095.1	'*Candidatus* Megaira', Clade A	PRJNA523022	Unknown	Freshwater	98.58	2.37	1657923	74	33	Contigs
JAFLDA01	GCA_017302595.1	'*Candidatus* Megaira', Clade E	PRJNA704939	Unknown	Activated sludge	99.53	0.95	1325166	3	34.5	Contigs
RFTG01	GCA_009923565.1	'*Candidatus* Megaira', Clade A	PRJNA495371	Unknown	Freshwater	68.27	3.28	918542	365	34	Contigs
NVVL01	GCA_002402195.1	n/a	PRJNA391950	Unknown	Marine	91.07	6	1905515	111	40.5	Contigs
JAIELT01	GCA_019752735.1	'*Candidatus* Megaira', Clade E	PRJNA745370	Unknown	Drinking water	56.92	1.34	897267	126	35	Contigs
RXKF01	GCA_003963235.1	n/a	PRJNA490743	Unknown	Freshwater	86.97	4.66	1361144	88	30.5	Contigs
JACCWQ01	GCA_013697555.1	'*Candidatus* Megaira', Clade E	PRJNA630822	Unknown	Soil	69.18	0.96	895 256	209	34.5	Contigs

### Phylogeny and evolution

**Fig. 1.**
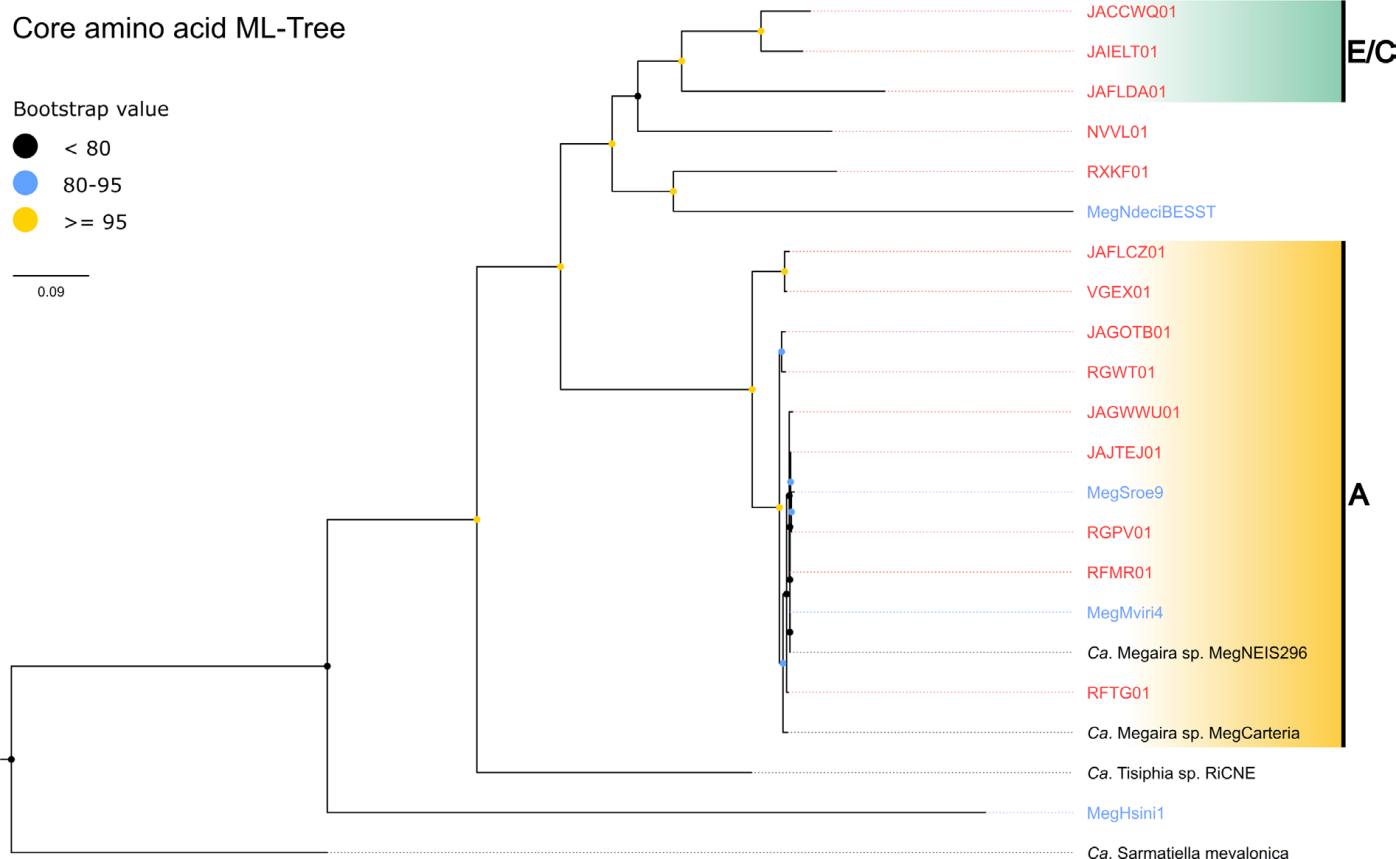
'*Ca*. Megaira' core genome maximum-likelihood tree based on 1000 ultrafast bootstraps. The scale bar = substitutions per site. Support for each split is shown as coloured circles, with strong support being ≥95. Samples from this study are shown in blue and existing environmental metagenomes in red.

ANI and AAI scores alongside phylogenetic analysis suggest that the whole of the *'Ca*. Megaira*'* genus is deeply divergent (Figs 1, 4 and S1). For instance, AAI scores between Clade A and Clade E are <65 % (Fig. 3) – where Clade refers to previously identified pseudo-species groups based on 16S phylogeny – and there is no synteny between MegNEIS296 and JAFLDA01, representatives of each group (Fig. S2). The existing *'Ca*. Megaira*'* clades do not sufficiently describe the diversity seen within the group and our genomic data suggest that the *'Ca*. Megaira*'* clade groups may represent different genera.

**Fig. 2.**
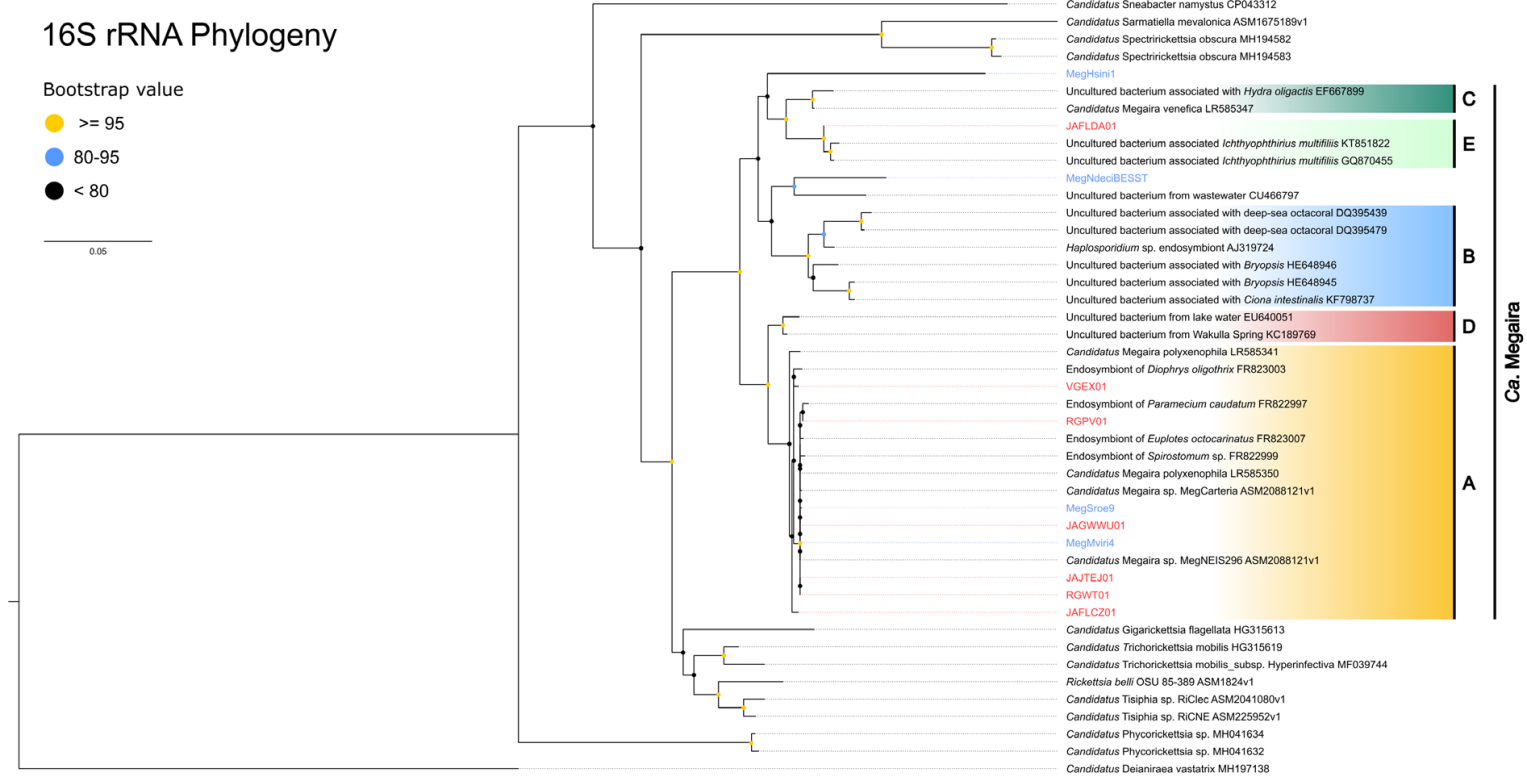
'*Ca.* Megaira' 16S rRNA maximum-likelihood tree based on 1000 ultrafast bootstraps. The scale bar = substitutions per site. Support for each split is shown as coloured circles, with strong support being ≥95. Samples from this study are shown in blue and existing environmental metagenomes in red.

**Fig. 3.**
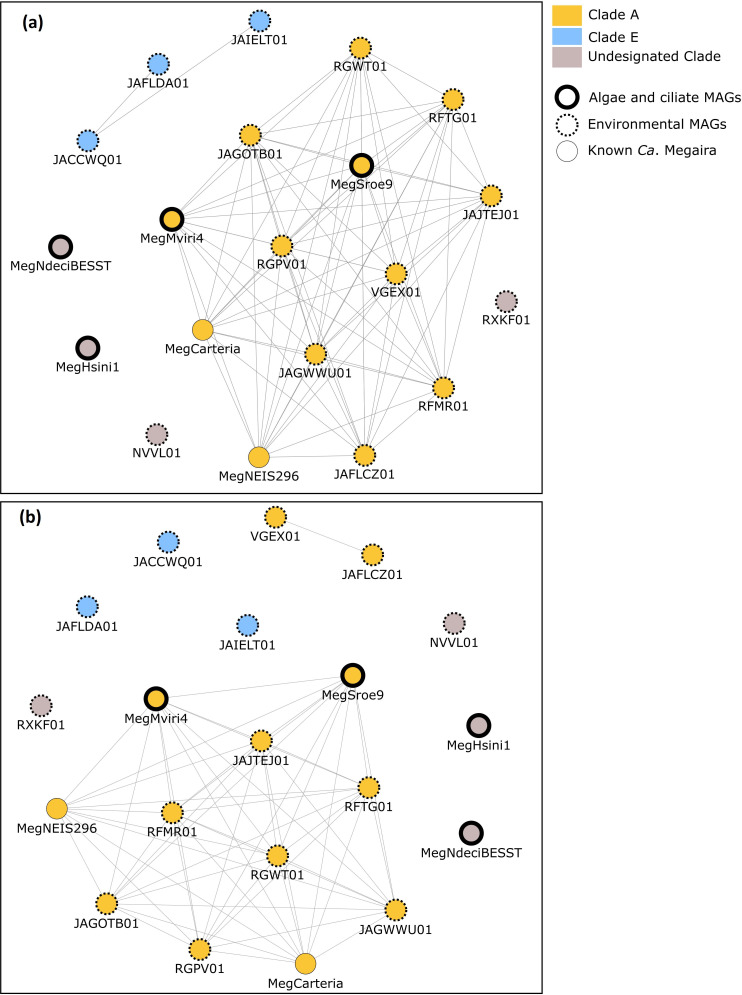
AAI and ANI map for '*Ca*. Megaira' showing (a) genomes sharing >65 % AAI similarity and (b) genomes with >95 % ANI similarity. Raw data can be found in Tables S3 and S4.

**Fig. 4.**
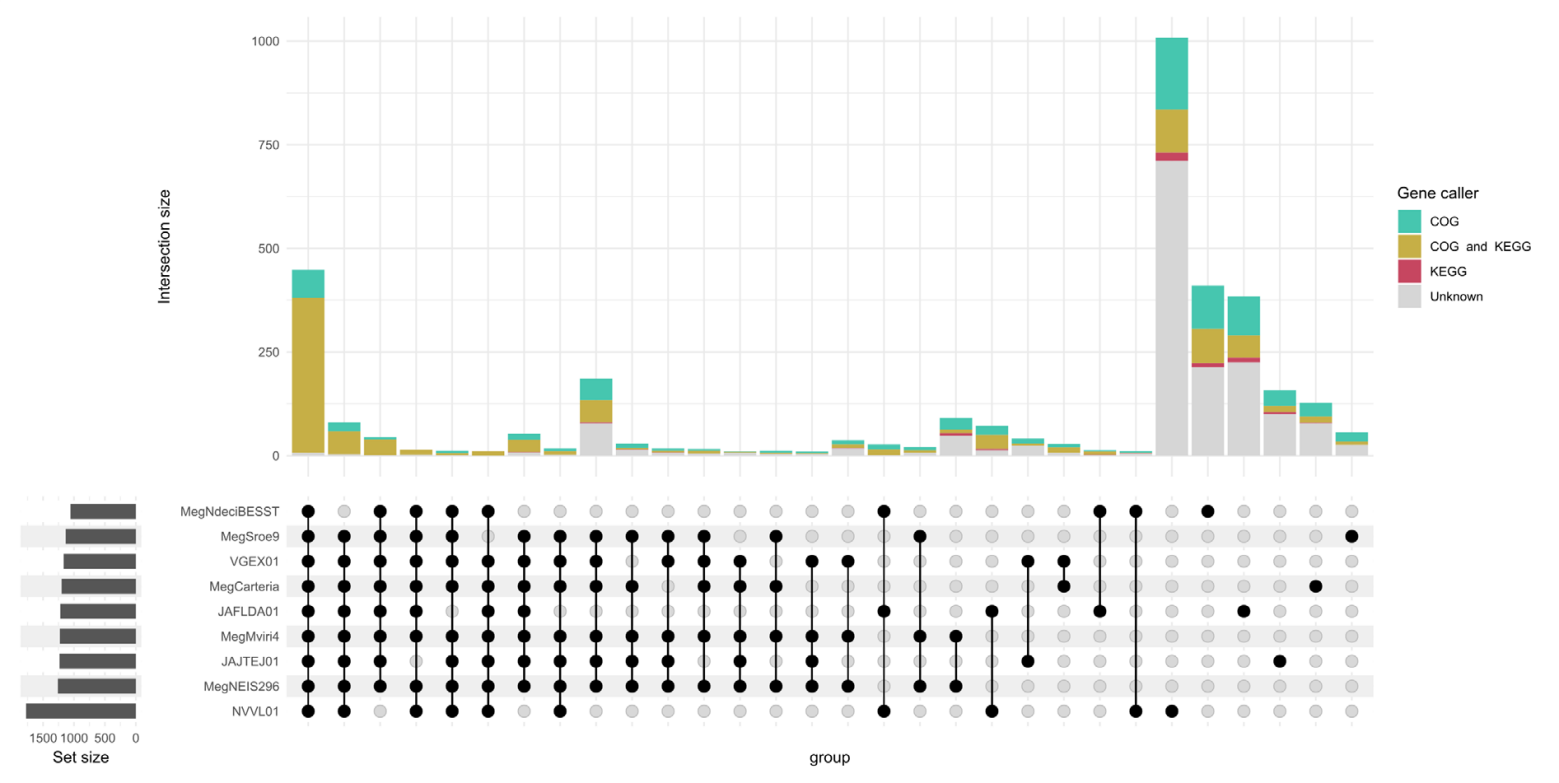
An upset plot showing the number of gene clusters (bars) shared between genomes ordered by intersection size and degree. Genomes being compared are indicated with black circles and lines. The number of known genes and the caller that identified them are indicated by bar size and colour. Presence–absence data can be found in Table S8.

Four of the *'Ca*. Megaira*'* draft genomes (MegHsini1, MegNdeciBESST, NVVL01 and RXKF01) represent new *'Ca*. Megaira*'* clades (Figs 1 and 2). AAI scores of <65 % suggest that these four are sufficiently derived to be considered new genera (Fig. 3). However, the placement of MegHsini1 within the *Rickettsiales* is currently uncertain (Figs 1, 2 and S1). For instance, GTDBtk classification does not assign MegHsini1 to a genus or species (Table S9). Based on available 16S rRNA and supporting AAI scores, most of the MAGs clustered within Clade A; three MAGs fall into Clade E (and possibly clade C); and two form a new group within Clade A which share an ANI similarity score of <95 % (Figs 2 and 3). Two MAGs lack 16S rRNA sequences and cannot currently be associated with any group as 16S rRNA is the only marker used to date to classify *'Ca*. Megaira*'*.

In several instances the genomes used in this study are the only ones available for their lineage (Fig. 1). In addition, MegHsini1 is very incomplete (62%) in comparison with the majority of others (11 of 18 are >85 % complete, Table 1), despite having high depth of coverage (~245×, Table S1). Although a 16S rRNA sequence was also recovered, MegHsini1 also is weakly placed in its phylogenies (Figs 1, 2 and S1) and potentially suffers from long branch attraction. At this stage we do not know if MegHsini1's uniqueness is a genuine feature or a symptom of fragmentation caused by amplification bias during the enrichment steps of single cell genomics. Further expansion of genomic data for *'Ca*. Megaira*'* is required to refine the phylogeny of the bacteria in this species, and we would recommend any future screening efforts use other indicator genes alongside 16S rRNA. The genomic information obtained here will enable development of these markers and PCR protocols.

Gene content analysis across the '*Ca*. Megaira' clades mirror these findings. The A group '*Ca*. Megaira' have a common shared unique gene set and have similar patterns of gene presence absence (Figs 4 and S1). Outside of clade A stains, NVVL01 is highly distinct, having over double the number of unique gene clusters compared to all other taxa; a large number of unique gene clusters were additionally observed in the other two non-A group strains, MegNdeciBESST and JAFLDA01 (Fig. 4).

### Metabolism, secondary compound synthesis, secretion systems and potential symbiosis factors

**Fig. 5.**
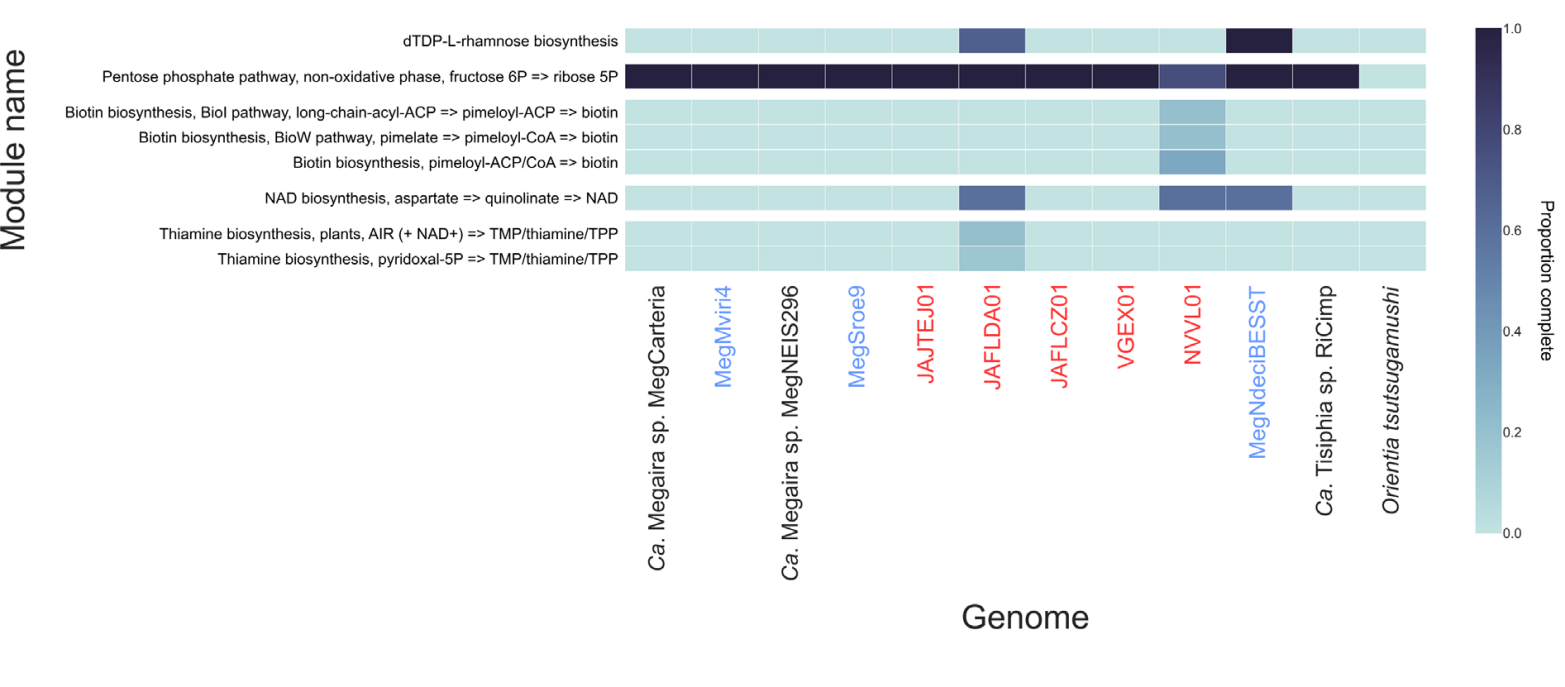
Metabolic heatmap of '*Ca*. Megaira', with '*Ca*. Tisiphia' RiCimp and *Orientia tsutsugamushi* as outgroups. KEGG Kofam module completeness from highest to lowest is shown with dark to light blue shading and pathways of interest are highlighted and circled with orange. Full metadata and additional pathways can be found in Table S6. Samples from this study are shown in blue and existing environmental metagenomes in red.

**Fig. 6.**
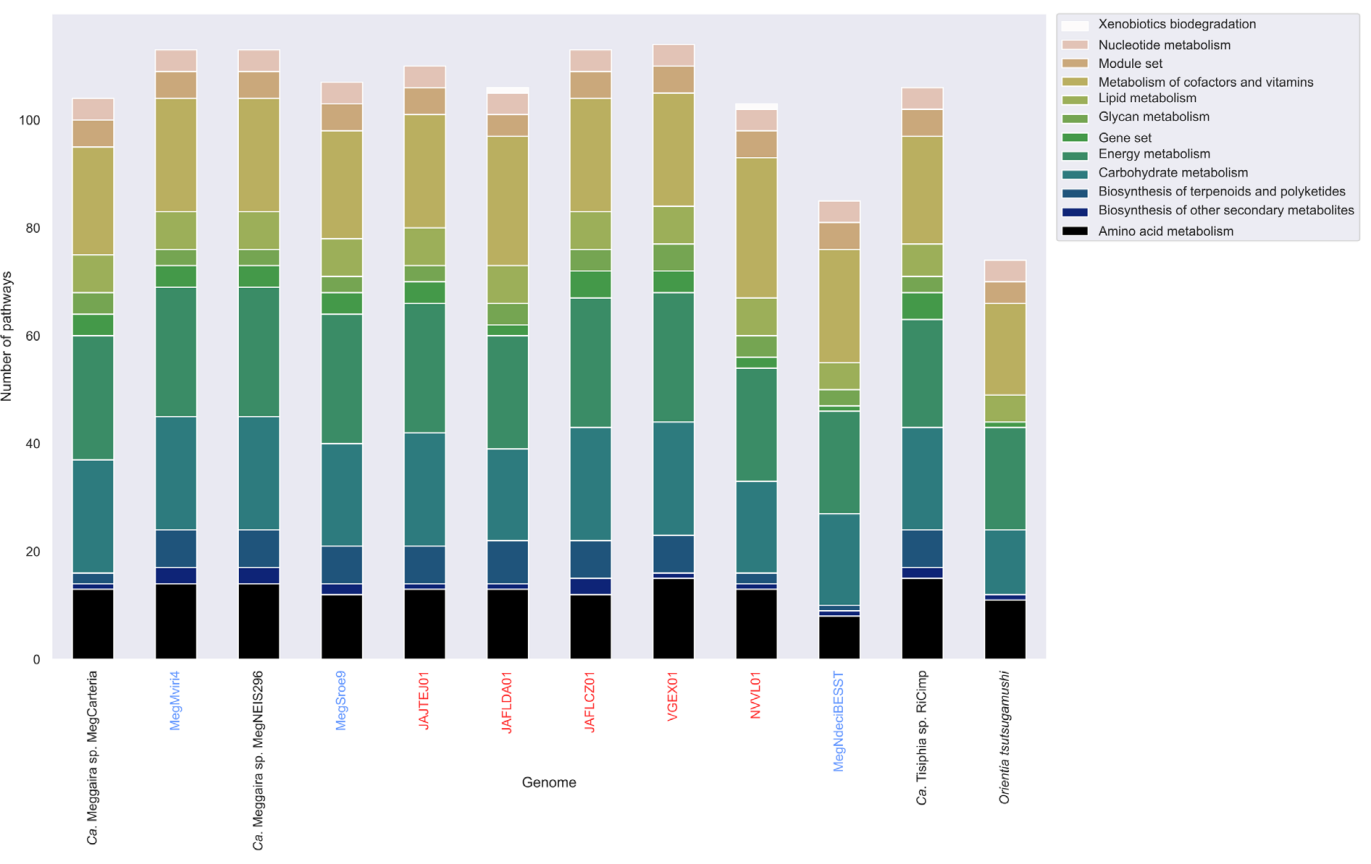
Number of pathways found per genome annotated by KEGG kofam module category for '*Ca*. Megaira', with '*Ca*. Tisiphia' RiCimp and *Orientia tsutsugamushi* as outgroups. Full metadata can be found in Table S5. Samples from this study are shown in blue and existing environmental metagenomes in red.

*'Ca*. Megaira*'* are not predicted to encode complete cofactor or vitamin pathways as would be typically observed in nutritional symbioses (Fig. 5, Tables S5 and S6). The genome JAFLDA01 is predicted to encode a partial thiamine pathway and NVVL01 a partial biotin biosynthesis pathway, neither of which are predicted to be functional without external inputs. All *'Ca*. Megaira*'* are predicted to have complete non-oxidative pentose phosphate pathways like their relatives, *'Ca*. Tisiphia' (=Torix Group *Rickettsia *) [32, 59]. MegNdeciBESST and JAFLDA01 have complete dTDP-l-rhamnose pathways (Fig. 5). Clade A *'Ca*. Megaira*'*, excluding MegCarteria, and clade E *'Ca*. Megaira*'* appear to be enriched for terpenoid and polyketide biosynthesis pathways compared to other taxa (Figs 5 and 6).

**Fig. 7.**
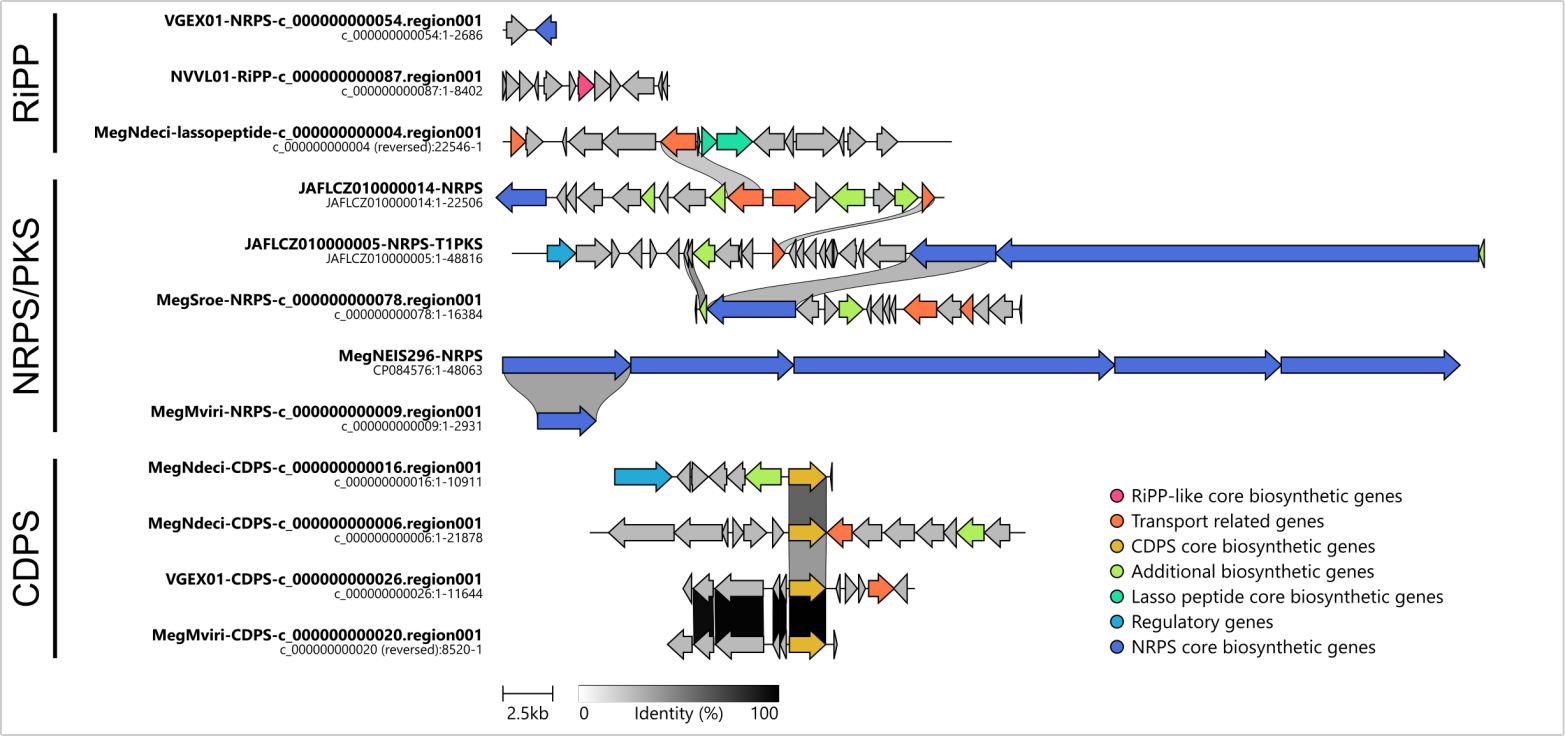
Clinker similarity diagram of RiPP, NRPS and CDPS gene regions found across '*Ca*. Megaira' by antiSMASH. Similarities between genes are indicated with grey shaded links between genes, and colours represent the types of genes present as found by antiSMASH. Rows are ordered by best overall similarity according to clinker defaults. A fully interactive clinker diagram with more details on each gene function can be found in Supplementary data.

AntiSMASH identified five putative NRPS/PKS (non-ribosomal peptide synthetases or polyketide synthases) systems in four of eight genomes examined (Fig. 7). It also predicted three predicted cyclodipeptide synthases (CDPS), and two ribosomally synthesized and post-translationally modified peptide systems (RiPPs), including one synthesizing a lasso peptide (Fig. 7, Supplementary data). Blastp found that the MegMviri4 contig containing the putative NRPS has 100 % similarity with the NRPS found previously in MegNEIS296, albeit it is only a partial fragment. Considering the highly repetitive structure of the NRPS modules, such systems are poorly assembled with only short reads. We also observed that MegMviri4 and VGEX01 share extremely similar CDPS systems (Fig. 7). Overall, according to blastp, the CDPS, NRPS and RiPP systems were most similar to those found in the two existing *'Ca*. Megaira*'* genomes, MegCarteria and MegNEIS296 (Table S10).

A mostly complete flagellar apparatus was also identified in JAFLDA01 (Table S5 and Fig. S3). Partial flagella pathways are also annotated in the genomes NVVL01 and RXKF01 (Table S5). Aside from these, *'Ca*. Megaira' strains all carry Sec and Tat systems for translocation of proteins to the periplasmic space, alongside one or more type IV secretion systems (Table S5).

We examined the *'Ca*. Megaira*'* genomes for ORFs with three classes of motif associated with protein–protein interactions considered important in symbiont–host interactions: ankyrin repeat domains, tetratricopeptide repeats and leucine-rich repeats. These gene sets were not generally common across *'Ca*. Megaira*'* (Table 2). However, the MegNDeciBESST genome was notably enriched, including 15 ORFs carrying ankyrin repeats, 20 with predicted tetratricopeptide repeat motifs and four with leucine-rich repeat genes. Two other strains, NVVL01 and JAFLCZ01, have modestly increased complements of ORFs in this class (Table 2).

**Table 2.** Number of ORFs in '*Ca*. Megaira' genomes containing putative protein–protein interaction domains as recognized in pfam searches

	ORF feature		
	Ankyrin domains	Tetratricopeptide repeats	Leucine-rich repeats
MegNdeciBESST	15	20	4
MegSroe9	1	0	1
MegCarteria	1	1	1
MegMviri4	1	3	1
MegNEIS296	1	4	1
NVVL01	9	7	2
JAFLDA01	4	3	0
JAFLCZ01	5	4	7
JAJTEK01	2	3	1
VGEX01	4	4	2

## Discussion

Advances in metagenomic methods and data-mining techniques are enriching our understanding of microbial symbiont diversity. The genus *'Ca*. Megaira*'* represents a common and hyperdiverse clade of intracellular symbionts associated with microeukaryotes and algae. Using a metagenomic approach, we have assembled draft genomes for four *'Ca*. Megaira*'* species. One of these genomes was assembled into a single scaffold using mate-pair reads. In addition, we identified 14 previously existing MAGs in GenBank derived from previous environmental metagenome projects [60–71]. Of these, five can be considered high quality (>90 % complete, <10 % contamination).

Our data indicate *'Ca*. Megaira*'* is diverse enough to be considered its own family within *Rickettsiales *. The available genomes for previously recognized clades of *'Ca*. Megaira*'* share AAI similarity below 65 % as well as very low synteny between the two most complete genomes JAFLDA01, *'Ca*. Megaira*'* Clade E and *'Ca*. Megaira*'* Clade A from *Mesostigma viride* (Figs 1, 4 and S2). In addition, NVVL01, while firmly positioned within *'Ca*. Megaira*'*, has an enormous number of unique and unclassified gene clusters that exceed all other genomes described here; this novelty indicates a potentially enormous scope for further genomic diversity within the *'Ca*. Megaira*'* clades. Our data also indicate a new species group within the current Clade A (Figs 1 and 3). Overall, the analysis of our current and limited genomic data suggest that the *'Ca*. Megaira' lineage consists of at least six genus-level clades and nine species.

Nevertheless, our understanding of *'Ca*. Megaira*'* genomic diversity remains limited, as we are unable to consolidate the taxonomy for single genomes that fall outside the main clades or that lack 16S rRNA gene sequences resulting from metagenomic assembly [72]. As such, whilst our data indicate taxonomic revision is necessary, we have chosen not to challenge current levels of taxonomic classification to avoid confusion while our knowledge of this family of bacteria is still relatively small. Instead, we encourage future studies to diversify the markers that they use for identifying *'Ca*. Megaira*'* beyond 16S rRNA, and to obtain greater genomic information, particularly beyond clade A strains, to allow firm resolution of *'Ca*. Megaira*'* genomic diversity to allow this revision.

All *'Ca*. Megaira*'* genomes obtained have similar predicted metabolic potential which match the two currently available genomes for this group (Fig. 5). Many algae depend on external sources of biotin, thiamine and cobalamin, including from bacteria [73]. However, apart from some partial B vitamin pathways in JAFLDA01 and NVVL01, there is little evidence of capacity for vitamin-dependent nutritional symbioses in these bacteria (Fig. 5 and Table S6). Although the external provision of intermediate metabolites could in theory complement an incomplete pathway, we currently have no evidence that this is the case in *'Ca*. Megaira*'*. Indeed, NVVL01 lacks both bioA and bioD genes, which makes the functionality of the whole pathway questionable.

Most Clade A strains encode a large number of proteins related to terpenoid and polyketide pathways (Figs 5 and 6). These are known to be associated with plant–mycorrhizal and sponge–alphaproteobacteria defensive symbioses [74]. Terpenes are also produced by algae for defence systems, and some red algae appear to be reliant on bacteria-like terpene pathways to do so [75]. Terpenoids and polyketides can also increase host tolerance to various environmental stresses, including pathogenic bacteria and heavy metal pollution [74, 76]. In addition, MegNdeciBESST, which was recovered from a brown alga genome project, has a complete dTDP-l-rhamnose biosynthesis pathway which can be associated with establishing symbiosis in plants [77, 78]. Therefore, it is possible that *'Ca*. Megaira*'* form a type of defensive symbiosis with their hosts. However, these terpenoids could alternatively be part of establishing infection in the host algae, rather than a defensive symbiosis because bacteria use them to produce components of their cell walls [79].

The presence of systems predicted to synthesize secondary metabolites (NRPS, CDPS and RiPPs, including a lasso peptide) provide additional evidence that *'Ca*. Megaira*'* could be involved in protective symbiosis, or a toxin–antitoxin system which can be associated with reproductive manipulation [80]. These peptide groups cover a wide variety of bacterial secondary metabolites, many of which are associated with antimicrobial, antifungal, antiviral and antibiotic properties [81, 82]; lasso peptides additionally show very high levels of tolerance to environmental extremes of temperature and pH [82]. Alternatively, the products of these systems could be actively harmful to the host as some of these molecules, such as the RiPP nostocyclamide, have been shown to have anti-algal properties [83]. It is currently unknown if these systems are functional or how the products might affect their hosts. However, they do seem to be common in *'Ca*. Megaira*'* as they are present in six of the eight genomes examined here.

Some intracellular symbionts deploy an array of proteins which interact with host proteins to modify host cellular systems and establish symbiosis. The most widely recognized of these is the expansion of genes carrying ankyrin domains in *Wolbachia* [84, 85]. MegNDeciBESST is evolutionary distant from other *'Ca*. Megaira*'* and has a clearly expanded repertoire of genes encoding ankyrin domains, tetratricopeptide domains and leucine-rich repeat domains which are associated with protein–protein interactions. This distinction probably makes the molecular basis of its symbioses distinct from that of the other strains. The MegNDeciBESST genome is particularly interesting, as it indicates that the expansion of potential effectors functioning through protein–protein interactions that is observed in *Wolbachia* is not unique and has independently evolved in other intracellular symbionts. This aspect of the MegNDeciBESST genome also supports the biological diversity of symbiosis that exists within the current clade *'Ca*. Megaira*'*.

We also found evidence for a complete flagellar apparatus in a clade D *'Ca*. Megaira*'*, JAFLDA01. Although *Rickettsiaceae* do not typically have flagella, microscopy results suggested the presence of a putative flagellar structure in *'Ca*. Megaira*'* venefica [20], a member of *'Ca*. Megaira*'* clade C. The apparatus is also present in a few related genera such as '*Ca*. Trichorickettsia' and '*Ca*. Gigarickettsia' [86]. The presence of flagellar genes in a deep member of the *Rickettsiaceae* [87] further suggests that a flagellar assembly apparatus might have been an ancestral feature of *Rickettsiaceae* that was subsequently lost from most of the lineages. We do not know if these pathways are functional, but it is notable that complete or near-complete sets of these genes are found in several *'Ca*. Megaira*'* species, while the majority of *Rickettsiaceae* lack them entirely.

In conclusion, *'Ca*. Megaira' is emerging as a diverse, cosmopolitan clade of bacteria that often form symbioses with a variety of ciliates, and micro- and macro-algae. It is commonly found in aquatic metagenomes [20] and is probably associated with many other microeukaryotes. We assembled four new draft genomes and identify 14 existing environmental MAGs. It is still unclear how these bacteria interact with their hosts, but the presence of partial terpene pathways, alongside the occurrence of various ORFs, NRPS, CDPS and RiPPs across *'Ca*. Megaira' could point towards defensive symbioses. We do not believe that the current taxonomy of *'Ca*. Megaira' sufficiently describes the diversity we observe here. However, further investigation is needed to fully consolidate the identity of genomes lacking 16S rRNA and increasing genome representation to avoid clades being represented by a single genome clade. Once this is complete, the diversity and biology of this hyperdiverse group can be established with greater power.

## Supplementary Data

Supplementary material 1

Supplementary material 2

Supplementary material 3
