# Supplementary material for: 'Candidatus Megaira' are diverse symbionts of algae and ciliates with the potential for defensive symbiosis
Source: Microb Genom. 2023 Mar 10;9(3):mgen000950. doi: 10.1099/mgen.0.000950 (PMC10132079; doi:10.1099/mgen.0.000950)
Supplement: Supplementary material 1 [file mgen-9-950-s001.pdf]

Supplementary figures for the paper:

'Candidatus Megaira' are diverse symbionts of algae and ciliates with the potential for defensive symbiosis

Helen Rebecca Davison, Gregory D. D. Hurst, Stefanos Siozios

Affiliation

Institute of Infection, Veterinary and Ecological Sciences, University of Liverpool, Crown Street Liverpool L69 7ZB UK

Corresponding author

Helen R. Davison, email: hlhdavi5@liverpool.ac.uk

a) Core amnio acid Bayesian phylogenetic inference

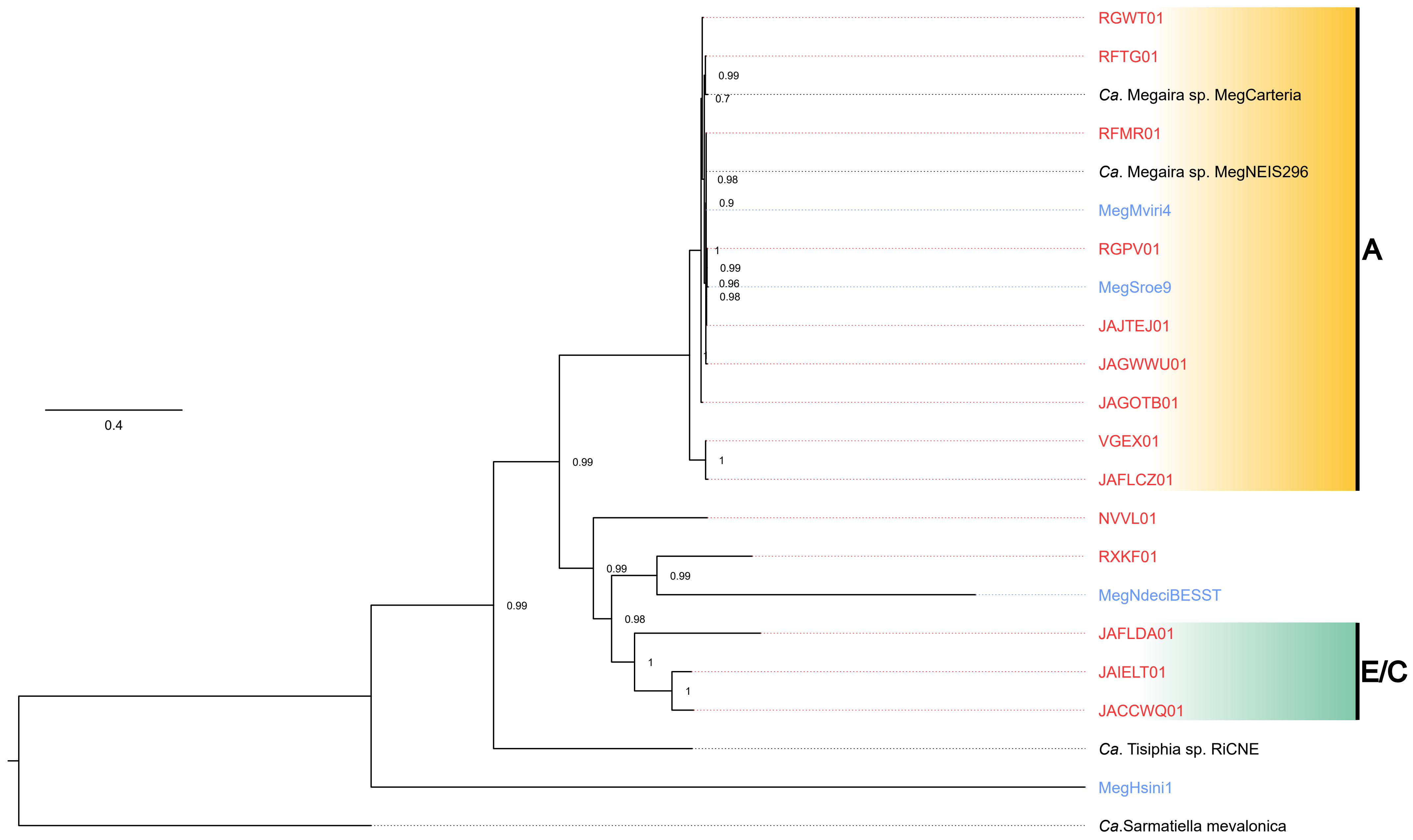

b) Gene cluster presence/absence phylogeny

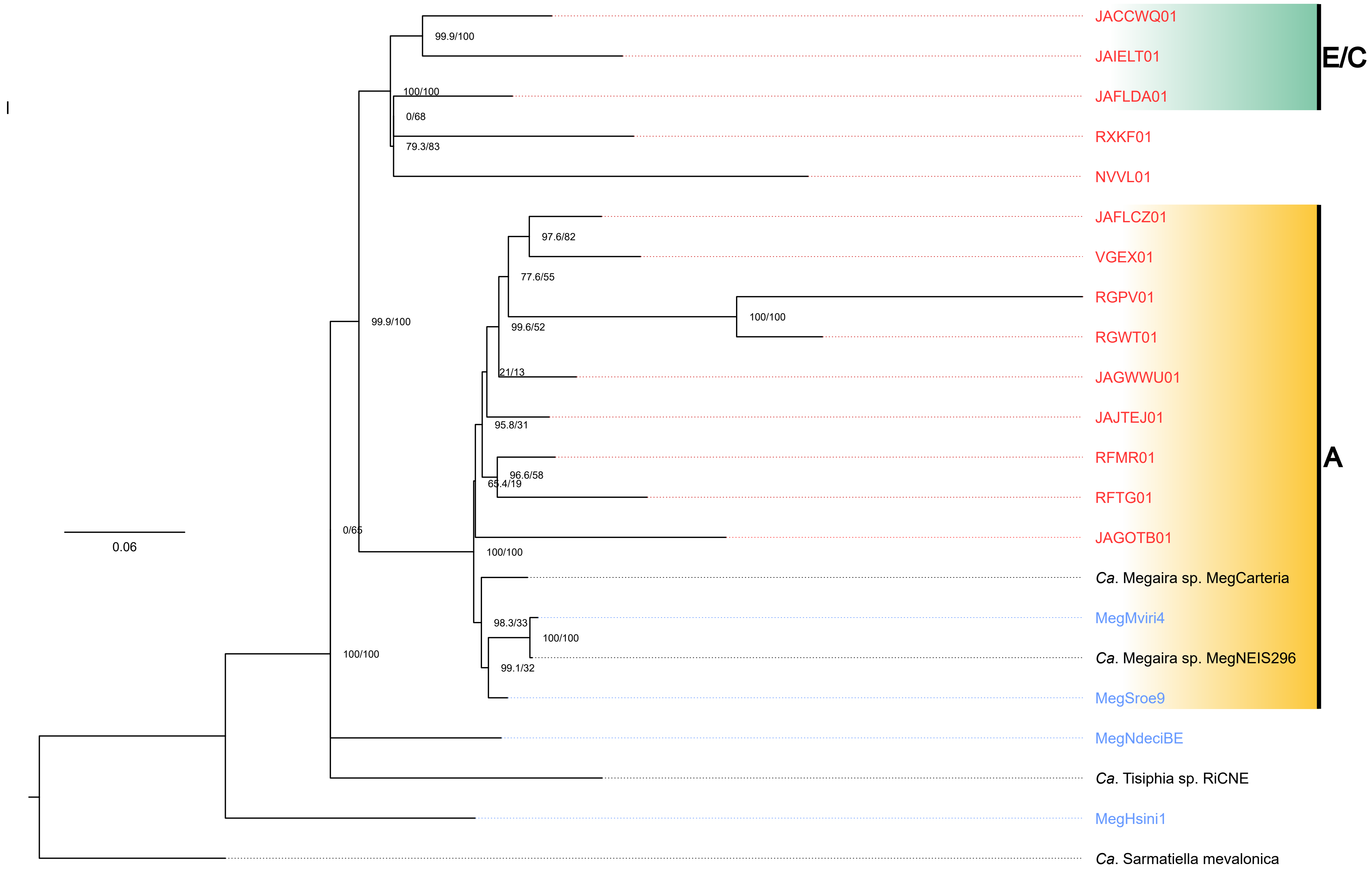

Supplementary Figure 1. Supporting '*Ca.* Megaira' phylogenies for main Figure 1. a) core amino acid Bayesian phylogenetic tree with nodes displaying posterior probability values, and b) a maximum likelihood tree of gene cluster presence absence with 1000 SH-aLRT and ultrafast bootstraps (UFB). Support for each split is displayed as SH-aLRT/UFB values, with strong support being >=80/>=95. Samples from this study are blue and existing environmental metagenomes are red.

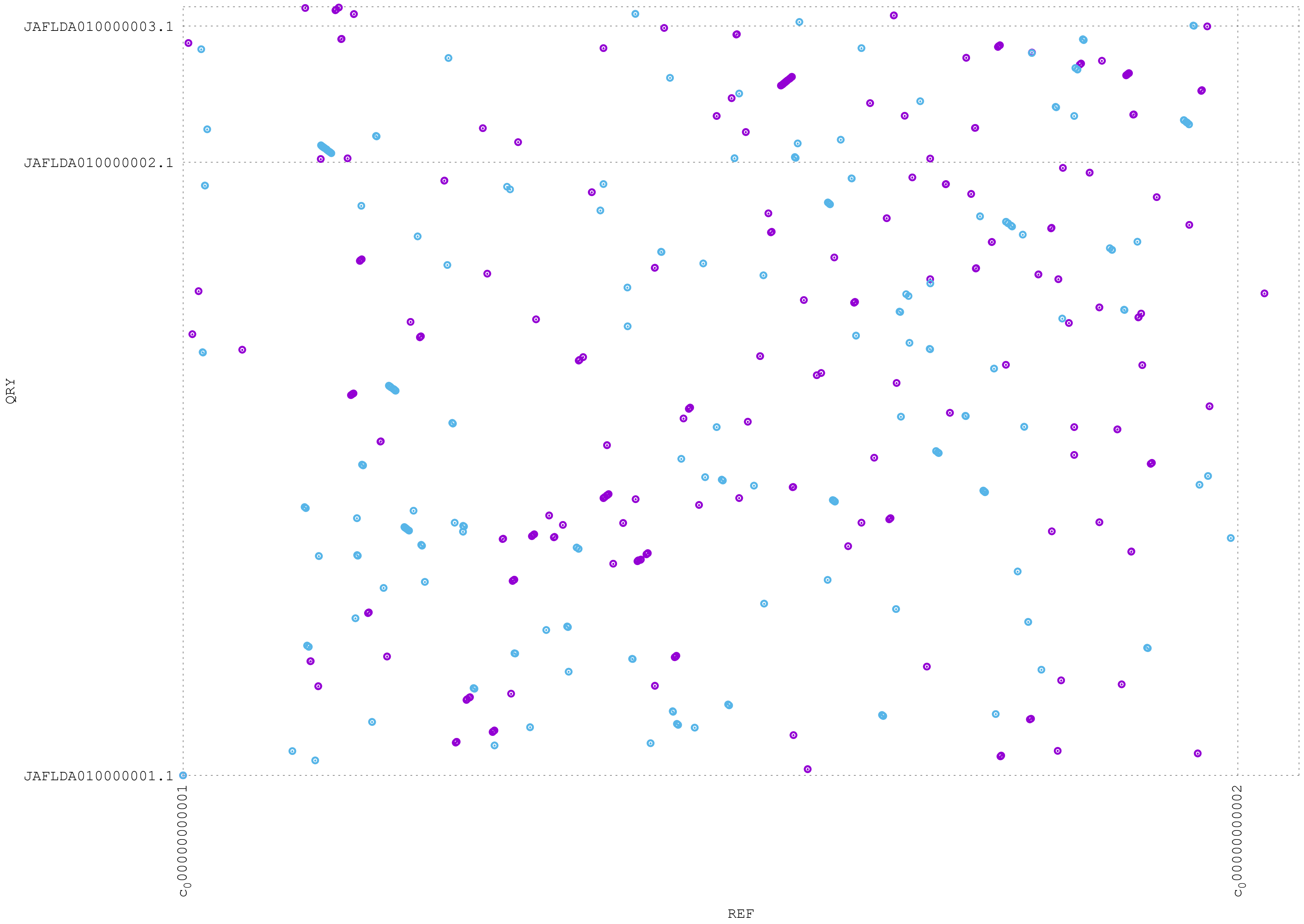

Supplementary Figure 2. PROmer synteny map constructed by mummer, comparing the similarities in patterns of protein loci across JAFLDA01 and MegNEIS296. Purple are forward matches; blue are reverse matches.

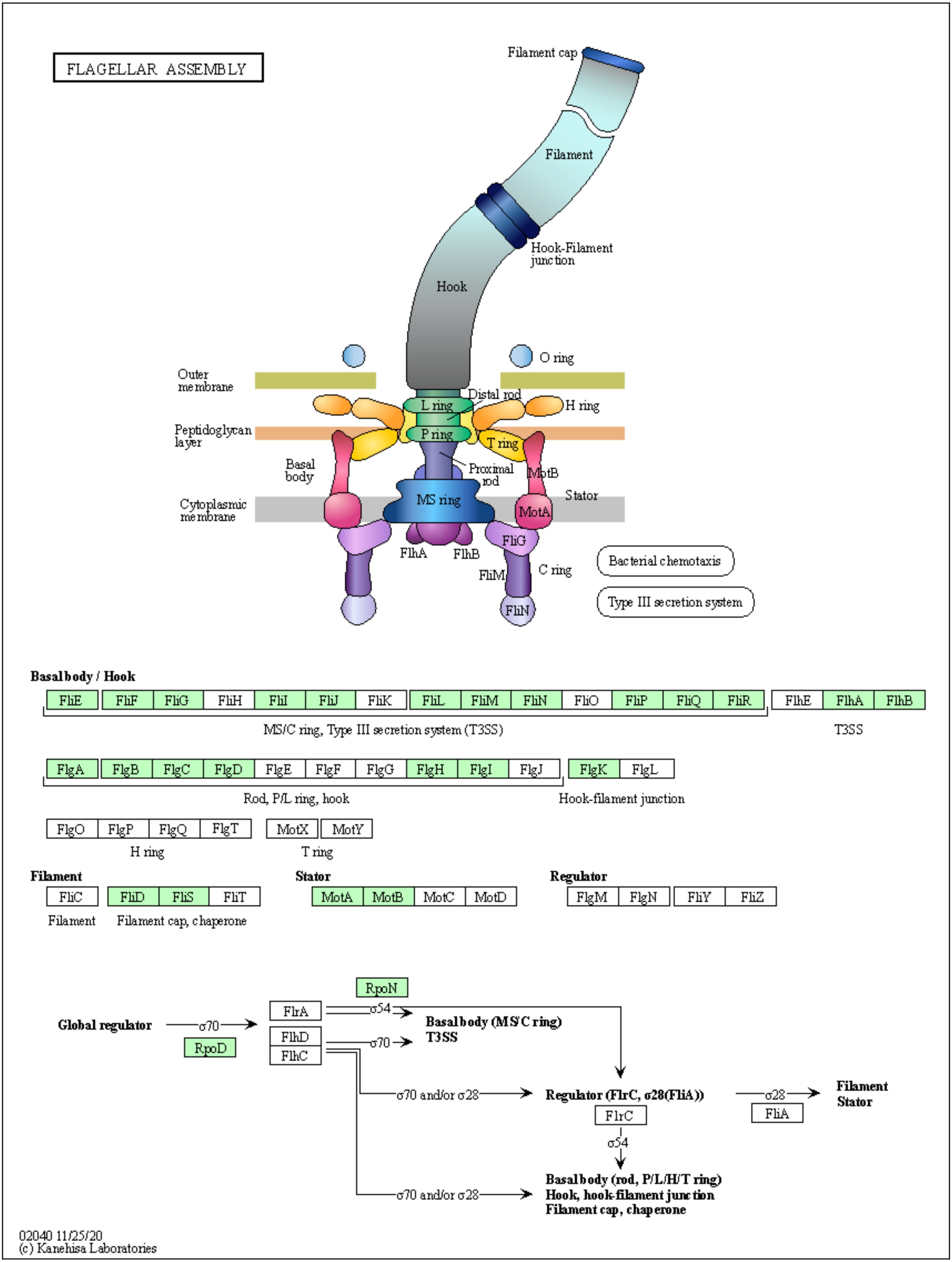

Supplementary Figure 3. Flagella apparatus for JAFLDA01. Green highlighted boxes indicated genes found.
