# Supplementary figures and images for: 'Candidatus Megaira' are diverse symbionts of algae and ciliates with the potential for defensive symbiosis
Source: Microb Genom. 2023 Mar 10;9(3):mgen000950. doi: 10.1099/mgen.0.000950 (PMC10132079; doi:10.1099/mgen.0.000950)

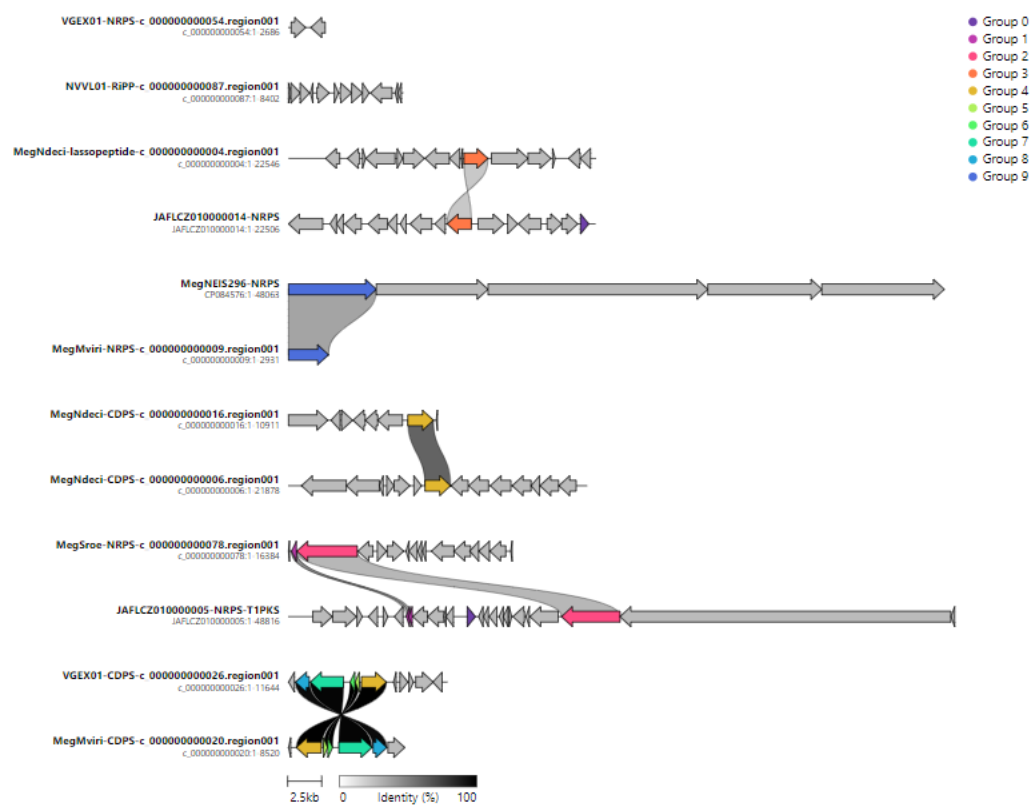

Supplement: Supplementary material 3 [file mgen-9-950-s003.pdf]
